# Utilisation of distally based sural fasciocutaneous flaps in lower extremity reconstruction: a single-centre experience with 88 paediatric patients

**DOI:** 10.1186/s13018-021-02206-x

**Published:** 2021-01-13

**Authors:** Zhaobiao Luo, Jiangdong Ni, Guohua Lv, Jianwei Wei, Lihong Liu, Ping Peng, Zhonggen Dong

**Affiliations:** 1 Department of Spine Surgery, The Second Xiangya Hospital, Central South University, Changsha, 410011 People’s Republic of China; 2 Department of Orthopaedics, The Second Xiangya Hospital, Central South University, Changsha, 410011 People’s Republic of China

**Keywords:** Lower extremity, Paediatrics, Soft tissue defect, Sural flap

## Abstract

**Background:**

No large series have analysed distally based sural fasciocutaneous (DBSF) flaps in paediatric patients. The aims of this study were to assess the reliability and analyse the potential risk factors for these flaps and to describe complications in the donor site and the functional follow-up results.

**Methods:**

Between June 2002 and November 2017, 88 DBSF flaps were used to reconstruct soft tissue defects in paediatric patients. Potential risk factors, reconstruction outcomes, and complications in the donor site of the flaps were analysed.

**Results:**

Among the 88 flaps, partial necrosis developed in 8 flaps (9.1%). The partial necrosis rate was significantly higher in flaps with the top edge located in the 9th zone (26.1%), with a length-width ratio (LWR) ≥ 5:1 (28.6%), and with a dimension of the skin island ≥ 100 cm^2^ (22.7%). Partial necrosis did not occur in flaps with a dimension of the skin island < 80.0 cm^2^ or with a skin-island width < 7.0 cm. The reconstruction outcomes in most paediatric patients were evaluated as “excellent” or “good”. The incidence of obvious scarring was higher in the donor site.

**Conclusions:**

Partial necrosis of DBSF flaps will significantly increase when the top edge of the flap is located in the 9th zone, when the LWR of the flap is ≥ 5:1, or when the dimension of the skin island is ≥ 100.0 cm^2^. Flaps with a skin-island width < 7.0 cm or with a dimension of the skin island < 80 cm^2^ are relatively safe and reliable.

## Introduction

Reconstruction of soft tissue defects in the distal leg, ankle, and foot is always a delicate problem, especially for paediatric patients. Many types of flaps can be utilised to reconstruct defects in the extremities of paediatric patients, such as local flaps, other pedicled flaps, cross-leg flaps, and free flaps, and each flap has its own respective characteristics and limitations [1–14]. Limited flap size and mobilisation are the drawbacks of local flaps and other pedicled flaps. Cross-leg flaps represent a relatively safer method for paediatric patients, but the legs need to be immobilised with plaster casts or with external fixation, and reoperation is unavoidable to remove the pedicle. Larger defects can be repaired by free flaps, and the donor sites of free flaps can be closed primarily at times, but a tedious surgical procedure is needed with a special microsurgical team, and there is always a risk of re-exploration [13–16].

Since distally based sural fasciocutaneous (DBSF) flaps were reported by Masquelet et al. [17], most authors have focused on applying these flaps in adults [18–26], and only a few authors have focused on the reconstruction of soft tissue defects in paediatric patients using these flaps [27–34]. Partial necrosis of DBSF flaps, which was associated with flap factors, was analysed in our previous study [35]. However, a minority of subjects in the study were children, with a majority of adults. There might be differences between the two groups in terms of potential flap risk factors. A specific focus on analysing potential risk factors for DBSF flaps with a considerable sample size for the reconstruction of these defects in paediatric patients is lacking. The aims of this study are to assess reliability and analyse the potential risk factors for DBSF flaps in paediatric patients with a considerable sample size and to describe complications in the donor site and functional follow-up results.

## Methods

All the legal guardians of the paediatric patients provided written informed consent to undergo the operation in combination with possible publication of the treatment and follow-up data. Approval was obtained from the ethics committee of Central South University. The procedures used in this study adhere to the tenets of the Declaration of Helsinki.

### Patients

Between June 2002 and November 2017, the clinical data of 88 DBSF flaps in 88 paediatric patients (age ≤ 12 years) for the reconstruction of soft tissue defects in the lower extremities were retrospectively reviewed and analysed. DBSF flaps were utilised because of soft tissue defects and exposure of vital structures in the lower extremities.

All the paediatric patients were evaluated preoperatively for their ability to tolerate the operation. If the paediatric patient was cooperative, the location of the peroneal artery perforator was mapped by colour Doppler ultrasonography. Additionally, none of the paediatric patients had a smoking history, peripheral vascular disease (PVD), or chronic medical disease, such as cardiovascular disease, high blood pressure, or diabetes mellitus (DM). The exclusion criteria were as follows: patients who were more than 12 years old; and patients who received cross-leg or proximally based sural fasciocutaneous flaps or perforator pedicled flaps.

The survival of flaps included those that completely and those with marginal necrosis. Flaps with marginal necrosis and those with partial necrosis had necrosis lengths less than 1.0 cm and more than 1.0 cm, respectively [36]. The potential risk factors for the flaps included patient factors and flap factors (i.e., width of the skin island, length-width ratio [LWR], location of the top edge of the flap, and so on), which were introduced in detail in a previous study [35]. The dimension of the skin island, which has rarely been noted previously, was also analysed in the present study. The shape of the skin island was usually irregular, the length multiplied by the width of the skin island was used to relatively accurately represent the dimension of the skin island, and the index indirectly represented the wound size.

### Operative technique

Radical debridement is one of the most important treatment measures to control infection. The skin island of the DBSF flap should be designed to be approximately 1.0 cm or 1.5 cm larger in the periphery than the form of the defect. The proximal edge of the flap should not extend beyond the popliteal fossa, and the lateral edges should not extend beyond the lateral midlines in paediatric patients. The DBSF flaps were harvested with the anterograde-retrograde method, which has been described in detail [37, 38]. The lesser saphenous vein and the sural nerve must be included as an axis line. To avoid compression of the subcutaneous tunnel and the pedicle, the flap was transferred to the defect through the incised passage. The donor site was closed either primarily or resurfaced with skin grafting. Postoperatively, elevation of the extremity was a useful measure to avoid compression and promote the venous drainage of the flaps. Hyperactivity of the paediatric patients was inevitable. In the hospital bed, strict limiting activities of the paediatric patients was unnecessary, and they were allowed to motion appropriately in the bed, but their parents were informed to look after carefully the patients to avoid the skin-island and fascial pedicle of DBSF flaps compression.

In addition, we evaluated the reconstruction outcomes according to the criteria described by Boyden et al [39]. The criteria included pain, appearance, footwear restrictions, functional restrictions, and patient satisfaction. A patient with “excellent” means that the patient reports no pain, no limitations in recreational or daily activities, and no footwear restrictions, and was satisfied. A patient with “good” means that the patient reports mild occasional pain, limitation of recreational but not daily activities, and no footwear restrictions, and was satisfied, with minor reservations. Complications in the donor site, including pigmentation and scarring, were also evaluated.

### Statistical analysis

Outcomes were analysed utilising the SPSS (version 17.0; SPSS Inc., Chicago, IL) statistical software package. Normal distribution was confirmed using the Kolmogorov–Smirnov test. The continuous variable data in the two groups are presented as the mean ± standard deviation or mean with range, and analysed by *t* test or Mann-Whitney *U* test. The categorical variable data are presented as constituent ratios and analysed by the chi-square test, or Fisher’s exact test. Multivariate logistic regression analyses were performed. Variables with a *p* values < 0.1 in univariate logistic regression analysis were entered into multivariate logistic regression analysis. Results with *p* values < 0.05 were considered significant.

## Results

The average time to elevate the flap was 32.6 ± 6.4 min. Of the 88 DBSF flaps, no flaps with complete necrosis were recorded, and 75 flaps survived uneventfully in one stage. Marginal necrosis occurred in five flaps, of which remanent defects were repaired by suturing in the second stage. Partial necrosis occurred in eight (9.1%) flaps. Of these eight flaps, only one flap required removal of the necrotic part and was repaired by transferring other regional flap, and the others were repaired by suturing (*n* = 2) or skin grafting (*n* = 5). The defects with the most distal edge located at the metatarsophalangeal joints were successfully covered.

Of the 88 paediatric patients, the average age of 67 males and 21 females was 6.9 years (range, 1–12 years). The main position of the soft tissue defects was the region of the Achilles tendon and heel. The aetiologies of the defects included traumatic (*n* = 82) and non-traumatic causes (*n* = 6); three defects were caused by unstable scars, two were caused by pressure sores on the heel, and one was caused by a tumour. The demographic data of all paediatric patients are shown in Table 1. The constituent ratios of the three indicators did not show any statistically significant differences between the flaps that survived and those with necrosis (*p* > 0.05).

**Table 1:** Comparison of demographic data between flaps that survived and flaps with partial necrosis

	Flaps that survived (*n* = 80) *N* (%)	Flaps with partial necrosis (*n* = 8) *N* (%)	***χ***^***2***^	***p***
Sex				0.390^*^
Male	62 (92.5)	5 (7.5)		
Female	18 (85.7)	3 (14.3)		
Position of defect			5.368	0.068
Pretibial region	3 (100.0)	0 (0.0)		
Achilles tendon and heel	50 (96.2)	2 (3.8)		
Dorsum of the foot	27 (81.8)	6 (18.2)		
Cause of defect				0.446^*^
Traumatic	75 (91.5)	7 (8.5)		
Non-traumatic	5 (83.3)	1 (16.7)		

^*^Fisher’s exact test

The continuous variable data of the DBSF flaps are shown in Table 2. The length of the skin island, total length of the flap, and dimension of the skin island were significantly greater in the flaps with necrosis (*p* < 0.05).

**Table 2:** Comparisons of continuous variables of flap parameters between flaps that survived and flaps with partial necrosis

Parameters*	Range	Flaps that survived (*n* = 80)	Flaps with partial necrosis (*n* = 8)	***U***	***t***	***P***^#^
Pivot point site (above tip of lateral malleolus) (cm)	3.5–11.0	6.11 ± 1.52	6.19 ± 1.46	306.5		0.839^#^
Fascial pedicle (cm)						
Length	2.0–9.5	5.68 ± 1.46	5.06 ± 2.64	276.5		0.523^#^
Width	2.5–5.0	3.75 ± 0.44	3.94 ± 0.18	244.0		0.205^#^
Skin island (cm)						
Length	5.0–17.0	9.58 ± 2.48	12.81 ± 2.14	91.0		**0.001**^#^
Width	4.0–13.0	7.96 ± 1.85	8.50 ± 1.28	264.0		0.414^#^
Total length (cm)	10.0–24.0	15.25 ± 2.80	17.88 ± 3.69		2.468	**0.016**^##^
LWR value	2.5–6.0	4.09 ± 0.73	4.55 ± 0.94	235.0		0.217^#^
Dimension of skin island (cm^2^)	30.0–165.0	78.00 ± 32.23	108.44 ± 22.18	127.5		**0.005**^#^

*The values are expressed as the mean ± SD

^#^ The Mann-Whitney *U* test

^##^*t* test

The categorical variable data of the DBSF flaps are shown in Table 3. When the top edge was located in the 9th zone or when the length-width ratio (LWR) of the flaps was 5:1 or more, the partial necrosis rate of the flaps increased significantly (*p* < 0.05). If the DBSF flap was harvested with a skin-island width ≥ 8.0 cm, the probability of partial necrosis increased, but no statistically significant differences were found between the groups (*p* > 0.05). Among 22 DBSF flaps with skin-island widths < 7.0 cm, no partial necrosis occurred. The dimensions of the skin island in the study ranged from 6.0 cm × 5.0 cm to 15.0 cm × 11.0 cm, and the average dimension was 80.8 cm^2^. The partial necrosis rate tended to increase with an increasing dimension of the skin island, especially in flaps with a dimension of the skin island ≥ 100.0 cm^2^. The partial necrosis rate in the DBSF flaps with a dimension of the skin island < 80.0 cm^2^ (0%, 0/45) was significantly lower than that in the flaps with a dimension of the skin island ≥ 80.0 cm^2^ (18.6%, 8/43) (Fisher’s exact test, *p* = 0.002). The partial necrosis rate in the DBSF flaps with a dimension of the skin island ≥ 100.0 cm^2^ (22.7%, 5/22) was significantly higher than that in the flaps with a dimension of the skin island < 100.0 cm^2^ (4.5%, 3/66) (Fisher’s exact test, *p* = 0.021).

**Table 3:** Comparisons of the constituent ratio of categorical variable data between flaps that survived and flaps with partial necrosis

	Flaps that survived (*n* = 80) *N* (%)	Flaps with partial necrosis (*n* = 8) *N* (%)	*χ*^2^	*p*
Pivot point (above tip of lateral malleolus)				0.678^*^
≤ 6 cm	59 (92.2)	5 (7.8)		
> 6 cm	21 (87.5)	3 (12.5)		
Top-edge location				0.004^*^
The 8th zone or lower zone	63 (96.9)	2 (3.1)		
The 9th zone	17 (73.9)	6 (26.1)		
Length-width ratio				0.020^*^
2.50 ≤ LWR < 5:1	70 (94.6)	4 (5.4)		
5 ≤ LWR ≤ 6:1	10 (71.4)	4 (28.6)		
Width of the skin island				0.459^*^
4.0 cm ≤ width < 8.0 cm	35 (94.6)	2 (5.4)		
8.0 cm ≤ width ≤ 13.0 cm	45 (88.2)	6 (11.8)		
Dimension of skin island			12.809	0.002
< 80.0 cm^2^	45 (100.0)	0 (0.0)		
80.0 cm^2^ ≤ dimension < 100.0 cm^2^	18 (85.7)	3 (14.3)		
≥ 100.0 cm^2^	17(77.3)	5(22.7)		

^*^Fisher’s exact test

In the study, independent factors associated with partial necrosis of DBSF flaps included the top-edge location (*p* = 0.047, odds ratio [OR]: 6.017, 95% confidential interval [CI]: 1.020–35.491), dimension of skin island (*p* = 0.045, OR 3.422, 95% CI 1.029–11.379). Sex, position and cause of defect, site of pivot point, width of the skin island, and LWR were not significant.

**Fig. 1:**
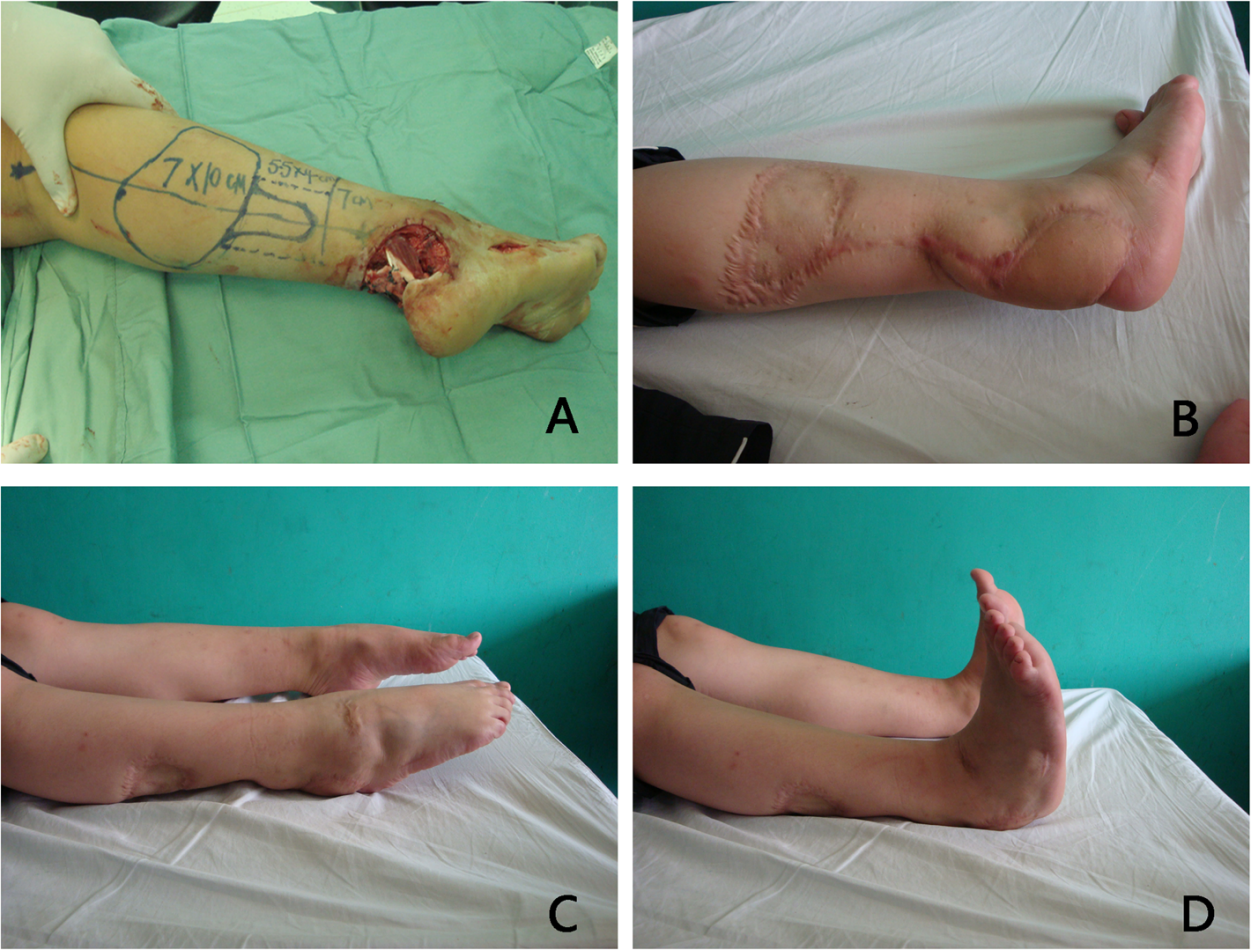
A 3-year-old girl suffered from a traumatic soft tissue defect in the Achilles tendon area. **a** Defect appearance after debridement, and a distally based sural fasciocutaneous flap was designed. **b** Appearance outcomes of the flap and donor site 21 months postoperatively. Pigmentation of the donor site did not occur, but obvious scarring of the donor site was observed. **c**, **d** “Excellent” reconstruction outcomes 21 months postoperatively

**Fig. 2:**
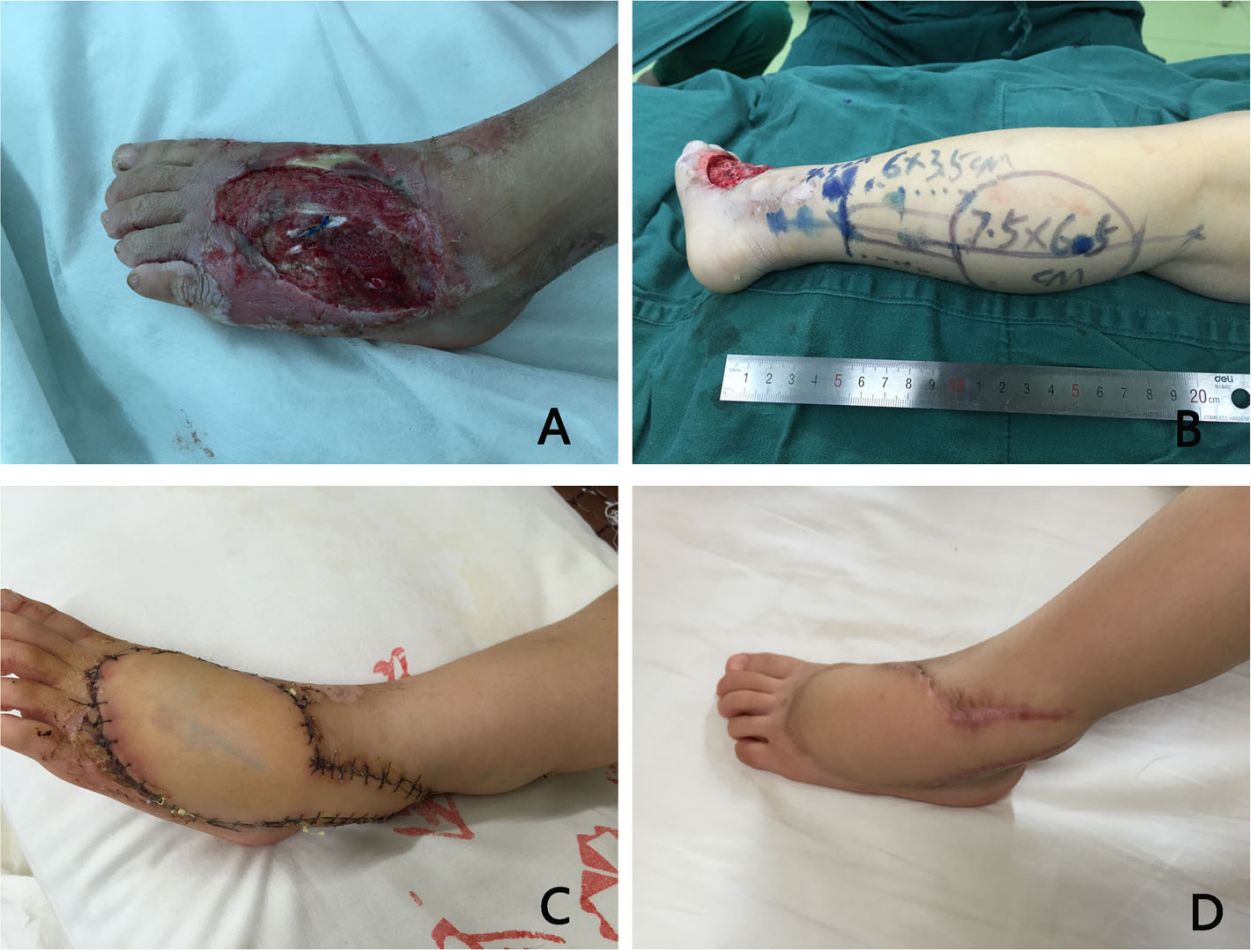
A 3-year-old girl suffered from a traumatic soft tissue defect in the dorsum area of the left foot. **a** Preoperative appearance of defect. **b**. A distally based sural fasciocutaneous flap was designed. **c** Appearance outcomes of the flap two weeks postoperatively, the flap survived uneventfully. **d** Appearance outcomes of the flap 9 months postoperatively

All the paediatric patients were followed, with an average period of 19.8 (range, 3–119) months. During the follow-up period, no infections recurred at the recipient site, three patients experienced ulceration due to poor shoe contact, and seven patients received reoperations because of debulking procedures. The visual gait analysis was nearly normal in most paediatric patients; only three patients had mild claudication, which did not hinder them from rapid walking, running, or participating in sports. In our series, no patients complained of insensitivity on the lateral aspect of the foot caused by the sacrificed sural nerve. The reconstruction outcomes of the paediatric patients were evaluated as “excellent” in 72 cases (Figs. 1 and 2), “good” in 14 cases, and “fair” in 2 cases. No patients were evaluated with “poor” outcomes. All the wounds of the donor site healed successfully, and no cases of infection or skin necrosis occurred at the donor site. The donor site was closed primarily in only five cases. Pigmentation of the donor site occurred in a few patients, but obvious scarring of the donor site was observed in the majority of paediatric patients. Scar contracture of the donor site did not affect the motion of the knee in the paediatric patients.

## Discussion

In DBSF flaps, flap viability-related complications, such as partial or complete necrosis, remain a concern. During the last decade or two, a few other authors reported the use of DBSF flaps in paediatric patients, and various necrosis rates were reported as follows: 0.0% (0/32) by Liu et al. [32], 5.0% (1/20) by Koladi et al. [28], 5.6% (2/36) by Zheng et al. [33], 12.5% (2/16) by Mahmood et al. [30], 12.5% (2/16) by Vergara-Amador et al. [29], and 20.0% (4/20) by Grandjean et al [34]. In the present study, the partial necrosis rate of the DBSF flaps was 9.1% (8/88). Seven remanent defects were covered successfully by using simple treatments. Only one remanent defect was covered successfully by transferring a regional flap. The average time to elevate the flap was approximately 30 min. The coverage range of the DBSF flaps in the study was from the lower leg to the metatarsophalangeal joints. The results of the study displayed the reliability, convenience, and large coverage range of DBSF flaps in paediatric patients.

Age, DM, PVD, and smoking are associated with flap failure [20, 26, 40]. However, all the patients in the study were paediatric patients, and none had histories of DM, PVD, or smoking. The probability of partial necrosis in DBSF flaps is associated with the width of the skin island, LWR, and location of the top edge [35]. In the present study, the potential risk factors for DBSF flaps were analysed emphatically. In the present study, when the top edge of DBSF flap was located in the 9th zone, the partial necrosis rate of the flaps significantly increased. This was similar to a previous paper [35]. The LWR of DBSF flap was not significant in the multivariate logistic regression analysis, but in the univariate analysis, when the LWR of DBSF flap was 5:1 or more, the partial necrosis rate of the flaps significantly increased. As introduced in the previous article [35], we considered that LWR ≥ 5:1 is also a risk factor for DBSF flap in the paediatric patients. However, a skin island width ≥ 8.0 cm was not a risk factor for DBSF flaps in paediatric patients. There was no partial necrosis of flaps when the width of the skin island was < 7.0 cm, although the maximum width of the skin island was 13.0 cm, and the flap successfully covered the defect in one stage. The results suggested that DBSF flaps with a width of the skin island < 7.0 cm are safer and more reliable for paediatric patients. According to Yang and Morris’s anatomic and angiographic study, the maximum width of the potential territory of the reversed sural island flap was 7.0 cm [18]. This may explain why the DBSF flap with a width of the skin island < 7.0 cm was safer and more reliable.

How large a flap can be for safe elevation in paediatric patients is controversial. Shea et al. reported that the wound surface area was a risk factor for flap complications in adult patients; flap procedures performed on larger wounds (size > 200 cm^2^) had approximately three times the overall complication rate than those performed on smaller wounds [41]. Various maximum dimensions of the skin island in paediatric patients have been described as follows: 5.0 cm × 9.0 cm by Mahmood et al. [30], 12.0 cm × 8.0 cm by Liu et al. [32], 8.0 cm × 10.0 cm by Koladi et al. [28], and 15.0 cm × 13.0 cm by Zheng et al. [33]. In their studies, the dimension of the skin island in the majority of the flaps was less than 80.0 cm^2^. Zheng et al. reported only seven flaps with a dimension of the skin island ≥ 100.0 cm^2^ [33]. In our study, the average dimension of the skin island was approximately 80.8 cm^2^, and the maximum dimension was 15.0 cm × 11.0 cm. The dimension of the skin island was 80.0 cm^2^ or more in approximately half of the patients, and the dimension was 100.0 cm^2^ or more in a quarter of the patients. No flaps had partial necrosis when the dimension of the skin island was < 80.0 cm^2^, and the partial necrosis rate in the flaps with a dimension of the skin island ≥ 100.0 cm^2^ was significantly higher. The results suggested that the flap could help repair relatively larger defects, the DBSF flap with a dimension of the skin island < 80.0 cm^2^ was safer and more reliable, and the possibility of partial necrosis increased significantly if the dimension of the skin island was ≥ 100.0 cm^2^.

In our study, the age of each paediatric patient was 12 or less 12 years old, and the average age of the paediatric patients was 6.9 years. In the paediatric population, the perforators originating from the peroneal artery are smaller in absolute size than those in adults [36]. Serletti et al. found vessel patterns in adolescent patients (ages between 13 and 17 years), such as size, wall characteristics, and spasm tendency, similar to those seen in adults, and the operative methods did not change compared with those in adults [1]. Herein, adolescent patients between 13 and 17 years of age were excluded from the study. In addition, trauma was the main aetiology leading to these defects (93.2%, 82/88). Motorcycle spoke injury and traffic accidents made up the majority of trauma cases in the study. The degree and complexity of injuries in paediatric patients are usually more serious [5, 31, 32]. The reasons might be parent negligence and the fact that the paediatric population is more likely to play on the street alone and have less vigilance to risk factors around them and that skin soft tissue has poor damage resistance ability [32].

The indications for and advantages of DBSF flaps have been described in detail in previous studies [25, 26, 28, 33, 34, 42]. In addition, another advantage is that injuries of adjacent structures can be synchronously reconstructed during DBSF flap surgery [31]. DBSF flaps have gained acceptance because of their advantages, but the major shortcoming of these flaps is the unsightly scar over the posterior calf, and some patients need debulking procedures to achieve aesthetics and comfort when wearing shoes. In the study, the donor site was not closed primarily because the subcutaneous fat in paediatric patients is thicker and the flaps were larger. Because subcutaneous fat in paediatric patients is thicker, the skin island of DBSF flaps should be designed to be approximately 1.0 cm or 1.5 cm larger in the periphery than the form of the defect. Otherwise, there will be tension in the stitching with the edge of the recipient site. Once tension is formed, the flap viability is affected.

During the follow-up period, the higher proportion of obvious scars in the donor site, the lower proportion of ulcerations in the flaps, and the few paediatric patients who required debulking procedures might have been related to the thicker subcutaneous fat and rapid body growth. The lower proportion of ulceration might be associated with the lighter body weight of paediatric patients and the partial sensory recovery [32]. The results of gait analysis and reconstruction outcomes were encouraging. This might be related to the higher adaptability in paediatric patients.

The main purposes for the reconstruction of defects in the lower leg, foot, and ankle are to restore lower extremity function, avoid amputation, control infection, and regain confidence by patients. Paediatric patients, as a special group, need to be specifically considered. Once an injury occurs in paediatric patients, it will affect them throughout their lifetime. Hu et al. assessed the psychosocial impact of multiple operations for paediatric patients, and the results indicated that the psychosocial functioning of paediatric patients was impaired if they underwent repetitive procedures [43]. Postoperative physical and psychological rehabilitation is beneficial to the normal development and healthy growth of paediatric patients [32]. In addition, paediatric patients grow up under the supervision of their parents. Hence, it is important for doctors to communicate with parents about the condition of paediatric patients.

The sample size of the study was considerable, and to the best of our knowledge, the study also has the largest sample size focusing on the application of DBSF flaps in paediatric patients at present. Because the study was a retrospective clinical study, a randomised controlled trial was not possible.

## Conclusions

The major shortcoming of distally based sural fasciocutaneous flaps is the unsightly scar over the posterior calf; however, these flaps represent an ideal option for paediatric patients to cover defects in the extremities because of their reliability, simplicity, and encouraging functional results. Partial necrosis of the flap will increase significantly when the top edge of the flap is located in the 9th zone, when the LWR of the flap is ≥ 5:1, or when the dimension of the skin island is ≥ 100.0 cm^2^. Flaps with a skin-island width < 7.0 cm or a dimension of the skin island < 80 cm^2^ are relatively safe and reliable. The outcomes will be helpful for flap planning to avoid partial necrosis in paediatric patients.

## Data Availability

Not applicable

## Abbreviations

DBSF: Distally based sural fasciocutaneous
DM: Diabetes mellitus
PVD: Peripheral vascular disease
LWR: Length-to-width ratio
OR: Odds ratio
CI: Confidential interval
